# Subretinal injection of balanced salt solution for macular edema secondary to retinal vein occlusion

**DOI:** 10.3389/fmed.2026.1701735

**Published:** 2026-02-26

**Authors:** Yue Pan, Xiang Zhang, Zetong Nie, Jiaxing Chi, Chang Liu, Qinning Xie, Mengqi An, Xiaorong Li, Wenbo Li, Bojie Hu

**Affiliations:** 1 Tianjin Key Laboratory of Retinal Functions and Diseases, Tianjin Branch of National Clinical Research Center for Ocular Disease, Eye Institute and School of Optometry, Tianjin Medical University Eye Hospital, Tianjin, China; 2 NHC Key Laboratory of Hormones and Development, Tianjin Key Laboratory of Metabolic Diseases, Chu Hsien-I Memorial Hospital and Tianjin Institute of Endocrinology, Tianjin Medical University, Tianjin, China

**Keywords:** anti-vascular endothelial growth factor, balanced salt solution, macular edema, pars plana vitrectomy, retinal vein occlusion, subretinal injection

## Abstract

**Purposes:**

To investigate the efficacy and safety of subretinal balanced salt solution (BSS) injections for macular edema secondary to retinal vein occlusion (RVO-ME).

**Methods:**

We retrospectively analyzed 19 eyes of 19 patients characterized by Retinal vein occlusion (RVO) diagnosed using retinal angiography and persistent or recurrent edema on optical coherence tomography after at least three standard-dose anti-vascular endothelial growth factor (VEGF) treatments. The operation group received pars plana vitrectomy (PPV) combined with internal limiting membrane (ILM) peeling and subretinal injection of BSS. The injection group continued to receive intravitreal injection of anti-VEGF drugs. The results of visual acuity, retinal morphology, and recurrence were analyzed and compared between the two groups after treatment. Statistical comparisons were adjusted for baseline imbalances using analysis of covariance.

**Results:**

After adjustment for baseline best corrected visual acuity (BCVA) and prior injection number, the difference in final BCVA between the surgery and injection groups was not statistically significant (*p* = 0.081). However, the surgery group demonstrated a statistically significant reduction in final Central macular thickness after adjustment (adjusted mean: 222.67 μm vs. 270.60 μm, *p* = 0.048). The surgery group also showed a strong trend toward a lower recurrence rate (40.0% vs. 88.9%, *p* = 0.051) and a longer median time to recurrence (10.0 vs. 4.0 months), though the latter was not statistically significant (log-rank *p* = 0.503). No serious adverse events were observed.

**Conclusion:**

This preliminary study suggests that PPV combine with ILM peeling and subretinal BSS injection was feasible and associated with significant anatomical improvement in refractory RVO-ME. The procedure has shown potential in controlling edema and reducing recurrence. While visual outcomes were comparable to anti-VEGF after adjustment, the anatomical benefit warrants further investigation in prospective trials.

## 1 Introduction

Retinal vein occlusion (RVO) is the second most common disease in vitreoretinopathy after diabetic retinopathy, and macular edema (ME) is the most common RVO complication, leading to visual impairment (1). The pathogenesis of macular edema secondary to retinal vein occlusion (RVO-ME) remains unclear and is mainly related to destruction of the inner blood–retinal barrier, high vascular permeability, and increased local inflammatory response (2). Clinically, the preferred treatment for RVO-ME is intravitreal injection of anti-vascular endothelial growth factor (VEGF) drugs, which have shown long-term therapeutic effects in restoring visual function and macular morphology (3). However, it has been observed in clinic and in some studies that the efficacy of anti-VEGF drug in ME treatment is limited in some patients, and the mechanism underlying this condition is complex. Notably, Ozurdex improves initial and refractory ME (4–6). Additional approaches such as cyst resection, combination therapy with Ozurdex and anti-VEGF agents, and subthreshold micropulse laser treatment have also been used for refractory ME (7–9). RVO-ME has been a very challenging issue for clinicians because it can lead to irreversible vision loss if not followed up and managed promptly, and some patients respond poorly to anti-VEGF therapy or experience recurrence after improvement.

Subretinal injection of a balanced salt solution (BSS) is a novel clinical technique which has been applied in some clinical studies, and its safety and effectiveness have been confirmed (10–12). Mao et al. used this technique to treat refractory diabetic macular edema (DME) and found that DME resolved significantly at 1 week and 1 month after surgery (13). Subretinal BSS injection may reduce the colloid osmotic pressure in the retinal tissue, remove inflammatory factors and migrating cells from the retinal pigment epithelium (RPE), and reduce the production and accumulation of inflammatory factors, thus improving persistent or recurrent edema To date, no studies have specifically examined subretinal BSS injection for RVO-ME. Considering the recurrent edema of RVO-ME and the burden of continuous injection, we considered whether surgery could achieve edema resolution, visual improvement, and long-term maintenance.

In this study, we investigated the efficacy of vitrectomy combined with subretinal injection of BSS compared with injection in the treatment of recurrent RVO-ME for edema resolution, visual improvement, and recurrence of edema.

## 2 Materials and methods

### 2.1 Study design

This was a single-center, retrospective, comparative cohort study conducted at Tianjin Medical University Eye Hospital. Patients with RVO-ME were enrolled from June 2023 through December 2024. This study was conducted in accordance with the principles of the Declaration of Helsinki and was approved by the Clinical Trials Ethics Committee of Tianjin Medical University Eye Hospital (2025KY-03).

### 2.2 Study population

**FIGURE 1:**
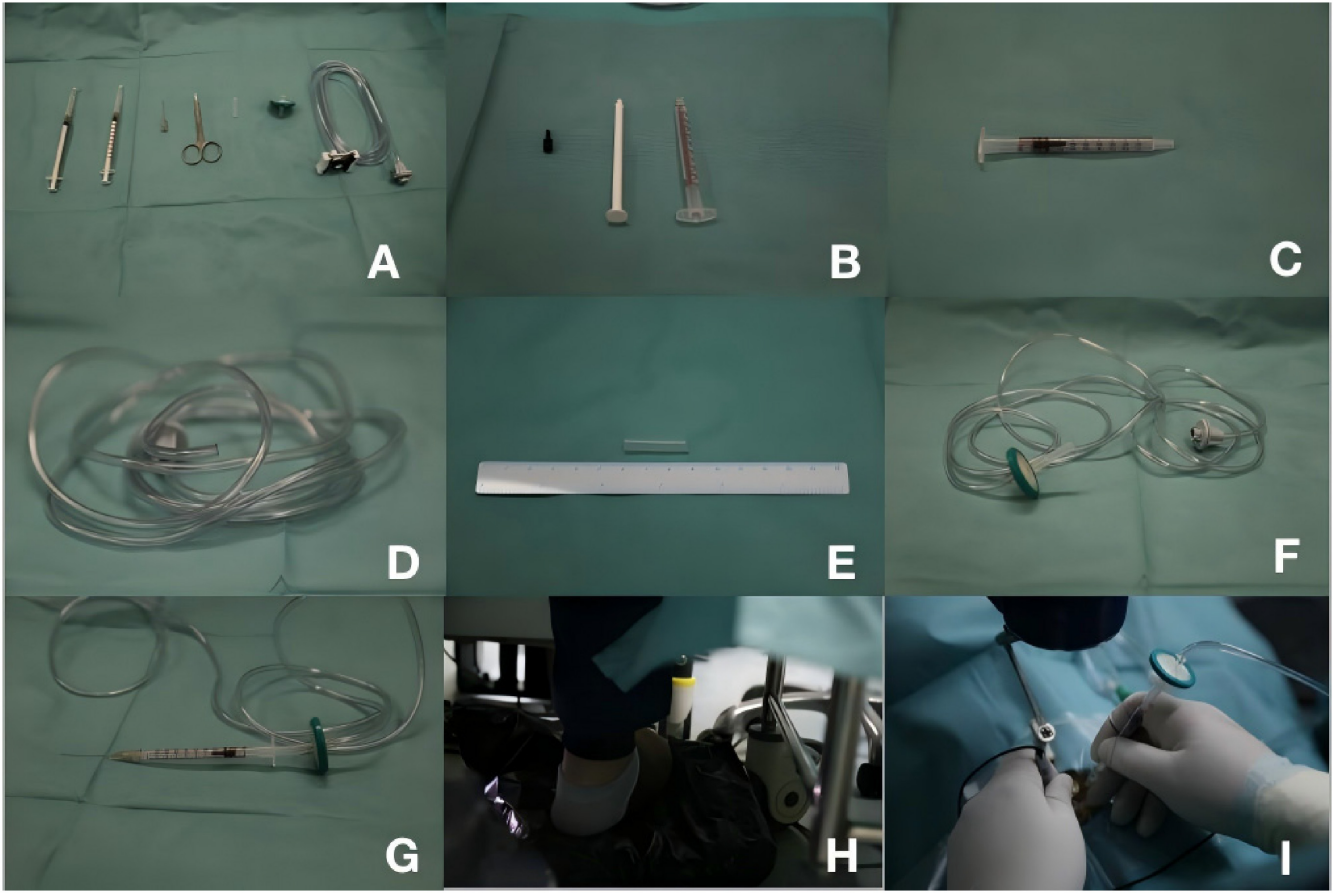
Assembly and application process of the novel subretinal injection device. **(A)** All the instruments required for assembling the device. **(B)** Removal of the plunger, plunger shaft, and plunger handle from the 1 mL syringe. **(C)** Re-insertion of the plunger into the empty cylinder of the 1 mL syringe. **(D)** Cutting the tubing connector from the Alcon^®^ viscoelastic control circuit. **(E)** Cutting a 3 cm-long silicone sleeve from the infusion tubing. **(F)** Connection of the needle filter to the cut Alcon@ viscoelastic control circuit and self-made silicone sleeve to form a preassembled device. **(G)** Connection of the 38G injection needle and preassembled device to the 1 mL syringe in the device. **(H,I)** Injection process using the device.

A retrospective review of medical records was conducted to identify consecutive patients with refractory RVO-ME at our institution between June 2023 and December 2024. The inclusion criteria were: (1) ≥ 18 years of age; (2) diagnosis of RVO on retinal angiography; (3) confirmed presence of ME on optical coherence tomography (OCT) images; and (4) persistent or recurrent edema after at least three standard doses of anti-VEGF therapy. Key exclusion criteria included: coexistence of other sight-threatening ocular pathologies (e.g., age-related macular degeneration, retinal detachment, or diabetic retinopathy), and insufficient follow-up duration (defined as < 3 months). A detailed flowchart of patient enrollment, outlining the numbers of eyes assessed, excluded, and included with specific reasons, is provided in Figure 1.

Following the screening process, a total of 19 eyes from 19 patients were eligible and included in the final analysis, 10 underwent surgery and 9 underwent intravitreal injections. The surgery group underwent vitrectomy combined with internal limiting membrane (ILM) peeling and subretinal injection of BSS. The injection group continued to receive anti-VEGF therapy.

### 2.3 Clinical study protocol

#### 2.3.1 Surgical procedure

All patients of surgery group underwent surgical procedures performed by the same experienced surgeon. If the patient had significant opacification, phacoemulsification and intraocular lens implantation were performed. The surgical procedure was conducted using an OPMI LUMERA T surgical microscope. The main surgical procedure was as follows:

A 25 gauge minimally invasive pars plana vitrectomy (PPV) was performed using the CONSTELLATION vitrectomy system, and complete posterior vitreous detachment was done with triamcinolone acetonide assistance. ILM approximately three optic disc diameters were peeling with the assistance of 0.025% indocyanine green staining. Subretinal injection of BSS was performed (see Subretinal Injection of BSS procedure). Air-liquid exchange was performed, and 1 mL of octafluoropropace (C3F8) was injected into the vitreous cavity. Following the surgery, all patients were instructed to maintain a prone position for 3 days to promote optimal recovery and absorption of the injected solution.

#### 2.3.2 Subretinal injection of BSS procedure

The device consists of two 1 mL syringes, a Millex^®^ 0.22μm needle filter, a set of Alcon^®^ viscoelastic material control tubing, a Medone^®^ 38G injection needle, and a custom-made, approximately 3 cm long, reusable silicone sleeve (Figure 1A). To assemble the device, first remove the plunger, plunger shaft, and plunger handle from one of the 1 mL syringes (Figure 1B). Then, reinsert the plunger into the empty barrel of the 1 mL syringe (Figure 1C). Cut off the connecting part of the Alcon^®^ viscoelastic material control tubing (Figure 1D). Cut a piece of silicone sleeve, approximately 3 cm long, from an infusion tube (Figure 1E). Connect one end of the needle filter to the cut Alcon^®^ viscoelastic material control tubing and the other end to the custom-made silicone sleeve to form a pre-assembled device (Figure 1F). Use another 1 mL syringe to extract BSS medication and transfer it to the empty barrel of the device’s 1 mL syringe. Connect the 38G injection needle and the pre-assembled device to the 1 mL syringe within the device (Figure 1G). Then, connect the device to the vitreous cutter’s silicone oil injection control unit and proceed with vacuum suction.

**FIGURE 2:**
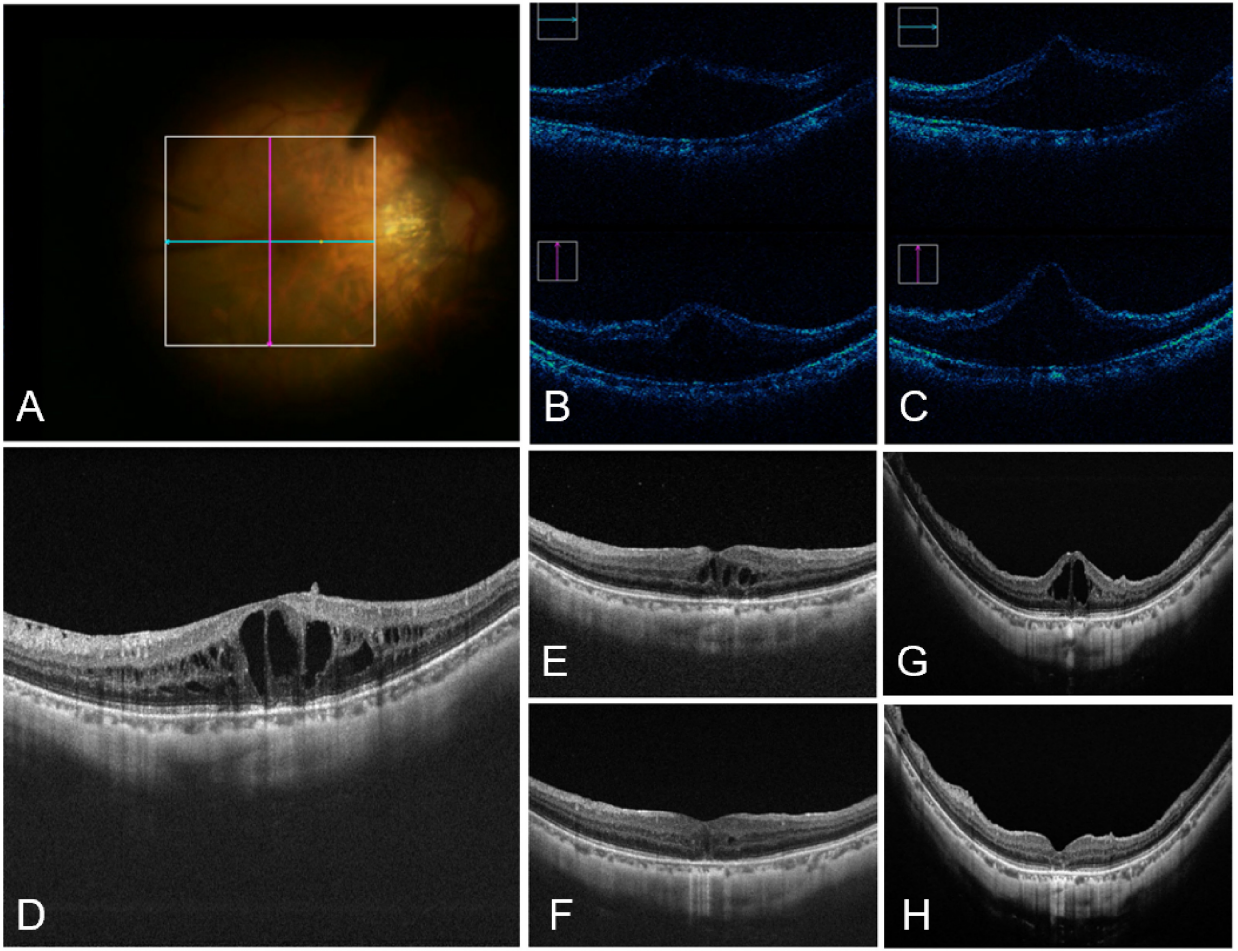
Intraoperative and postoperative images of one case in surgery group. **(A)** The fundus as seen in an intraoperative video and the area scanned by iOCT. **(B)** iOCT images of the corresponding regions before subretinal injection. **(C)** iOCT images of the corresponding regions after subretinal injection. The macular area was elevated, and no macular hole was observed. **(D)** Preoperative OCT images. **(E)** OCT images 1 week after surgery. **(F)** OCT images 6 months after surgery. **(G)** Cataract and recurrent ME were found during follow-up, and cataract surgery and anti-VEGF drug treatment were performed. **(H)** OCT images 2 months after cataract surgery and retreatment. ME resolved and the patient’s logMAR BCVA returned to 0.3.

Intraoperative OCT (iOCT; Zeiss RESCAN 700 OCT System, Germany) was used for real-time guidance. A 38G injection needle was inserted into the retina approximately 2–3 disc diameters away from the fovea. During the injection process, the pressure for injecting silicone oil was reduced to 6 psi. The injection speed was gently adjusted by pressing the foot pedal. When the foot pedal was depressed, constant pressure was applied to the plunger in the 1 mL syringe, causing the plunger to move forward (Figures 1H,I) and allowing controlled injection up to 0.05 mL of BSS into the subretinal space (Figure 2 and Supplementary Video 1, which demonstrates the surgical procedure and iOCT for subretinal injection).

#### 2.3.3 Intravitreal injection procedure

The patients were treated with intravitreal injection of anti-VEGF when the edema remained or recurred. Under surface anesthesia, drug injection was performed at the 11 o’clock position of the operative eye. After operation, the injection point was massaged with a cotton swab, and the patients were in free position.

#### 2.3.4 Intervention for recurrence

The injection group was treated with pro re nata therapy. Both groups were treated with anti-VEGF injections at follow-up when recurrent edema was noted. Recurrent edema was defined as: (1) existence of recent or persisting cystoid retinal lesions;(2) The patient had a decrease in best corrected visual acuity (BCVA) of more than two lines; and (3) an increase of 50 μm or more in central macular thickness (CMT) compared with the best value previously achieved.

### 2.4 Data collection

All participants were assessed prior to treatment, including the BCVA assessment, intraocular pressure measurement, slit-lamp microscopy, and spectral domain optical coherence tomography (SD-OCT). BCVA was evaluated using the international standard visual acuity chart, and the results were converted to the logarithm of the minimum resolution Angle (logMAR) visual acuity. A manual conversion was performed to convert Snellen visual acuity to 2.3 logMAR and counting fingers to 2.0 logMAR. CMT were measured using an SD-OCT device (Topcon Triton, Japan).

Slit-lamp and OCT examinations were performed in both groups during follow-up. BCVA, IOP and adverse complications were recorded. Recurrence as well as retreatment maintenance were collected.

### 2.5 Statistical analysis

Statistical analysis was performed with SPSS 26.0 (SPSS Inc., United States). Continuous variables were presented as mean ± standard deviation or median (interquartile range) based on their distribution. Categorical variables were summarized as frequencies and percentages (n,%). A normality test was used for all variables. Parametric tests were used to compare normally distributed data, while non-normally distributed data were compared using non-parametric tests. The paired *t*-test and the Wilcoxon signed-rank test were applied to evaluate changes in OCT and BCVA in the same group. Independent samples *t*-test and the Mann–Whitney test were used to compare baseline data and treatment effects between the two groups. The BCVA was analyzed using an ANCOVA model adjusted for baseline BCVA and number of previous anti-VEGF injections. The CMT was analyzed using an ANCOVA model adjusted for baseline CMT and number of previous anti-VEGF injections. The results of the ANCOVA are reported as adjusted means with their 95% confidence intervals (95% CI), the F-statistic, *p*-value, and partial eta-squared (partial η^2^) as a measure of effect size. Chi-square test was performed for recurrence conditions. A Kaplan-Meier survival analysis was used to assess the recurrence-free survival rate, with comparisons between groups made using the log-rank test. *P* < 0.05 was considered statistically significant.

## 3 Results

### 3.1 Baseline information

A total of 19 eyes from 19 patients were included in the final analysis and categorized into two groups: the surgery group (*n* = 10) and the injection group (*n* = 9). Patients were followed up at 1 week, 1 month, 3 months and every 3–6 months as needed. Median follow-up was 6 months (range 3 to 36). The baseline demographic and clinical characteristics of the two groups are shown in Table 1. The two groups were well-balanced in terms of age, gender, laterality of the affected eye, systemic comorbidities (diabetes and hypertension), and the distribution of RVO subtypes (all *p* > 0.05). However, significant baseline imbalances were observed in key ocular parameters. The surgery group presented with a significantly worse mean baseline BCVA (1.06 ± 0.31 logMAR) compared to the injection group (0.60 ± 0.28 logMAR; *p* = 0.004). Patients in the surgery group had received more previous anti-VEGF injections (4.10 ± 0.69) than those in the injection group (3.00 ± 0.00; *p* = 0.039). There was no statistically significant difference in baseline central macular thickness between the groups (*p* = 0.363).

**TABLE 1:** Baseline characteristics of the two groups.

Characteristic	Surgery Group (*n* = 10)	Injection Group (*n* = 9)	*P*-value
**Demographics**			
Age (years)	67.10 ± 10.90	67.33 ± 7.12	0.967
Gender (male/female)	2\8	4\5	0.350
Eye (right/left)	6\4	5\4	1.000
**Systemic comorbidities**			
Diabetes [n(%)]	3(30.0)	2(22.2)	1.000
Diabetes duration (years)	12.00 ± 15.62	7.5 ± 2.54	0.673
Hypertension [n(%)]	7(70.0)	6(66.7)	1.000
Hypertension duration (years)	9.87 ± 8.15	12.17 ± 15.33	0.735
**Ocular characteristics**			
RVO subtype [n(%)]			
CRVO	4(40.0)	6(66.7)	0.370
BRVO	6(60.0)	3(33.3)	
Baseline BCVA (logMAR)	1.06 ± 0.31	0.60 ± 0.28	0.004
Baseline CMT (μm)	512.50 ± 72.99	461.56 ± 154.60	0.363
Previous anti-VEGF injections	4.10 ± 0.69	3.00 ± 0.00	0.039

Data are presented as mean ± standard deviation, n (%), or n/n as appropriate. BCVA, best-corrected visual acuity; CMT, central macular thickness; CRVO, central retinal vein occlusion; BRVO, branch retinal vein occlusion; anti-VEGF, anti-vascular endothelial growth factor. *P*-values were calculated using independent *t*-test for normally distributed continuous variables and Fisher’s exact test for categorical variables.

### 3.2 Vision outcomes

The comparison of vision results is shown in Table 2. In unadjusted analysis, best BCVA did not differ significantly between groups (*p* = 0.622). And the surgery group showed a mean improvement in BCVA of 0.45 ± 0.40 logMAR from baseline to final visit (*p* = 0.006), while the injection group improved by 0.06 ± 0.27 logMAR (*p* = 0.160). The between-group difference in this change was statistically significant in unadjusted analysis (*p* = 0.025).

**TABLE 2:** Comparison of BCVA between the two groups.

BCVA measure	Surgery group (*n* = 10)	Injection group (*n* = 9)	*t*/*F*-value	*P*-value
**Unadjusted analyses**				
Baseline BCVA (logMAR)	1.06 ± 0.31	0.60 ± 0.28	3.387	0.004**
Best BCVA (logMAR)	0.60 ± 0.45	0.53 ± 0.49	0.493	0.622
ΔBCVA (logMAR)^†^	0.45 ± 0.40	0.06 ± 0.27	2.460	0.025*
Within-group *P*-value^‡^	0.006**	0.160	–	–
**Adjusted analysis (ANCOVA)**				
Adjusted best BCVA (logMAR) [95% CI]	0.787 [0.482, 1.091]	0.374 [0.089, 0.659]	3.502	0.081
**Covariates in ANCOVA model**				
Baseline BCVA			12.898	0.003**
Previous injections			0.081	0.780

Data are presented as mean ± standard deviation unless otherwise indicated. ANCOVA model *R*^2^ = 0.468. BCVA, Best-corrected visual acuity (expressed as log MAR); 1BCVA, Difference in BCVA before and after the intervention;

† Unadjusted between-group comparison by independent *t*-test;

‡ Within-group comparison by paired *t*-test.

*P*-value: *P < 0.05, **P < 0.01.

However, given the significant baseline imbalance in BCVA between groups, we performed an analysis of covariance adjusting for baseline BCVA and number of previous anti-VEGF injections. After adjustment for baseline BCVA and the number of previous anti-VEGF injections, the difference between the surgery group and the injection group was not statistically significant [mean difference = 0.412 logMAR, 95% CI: −0.057 to 0.882, *F*(1, 15) = 3.50, *p* = 0.081]. Baseline BCVA was a significant independent predictor of the final visual outcome [*F*(1, 15) = 12.90, *p* = 0.003], whereas the number of previous injections was not [*F*(1, 15) = 0.08, *p* = 0.780].

### 3.3 Edema outcomes

The outcomes for CMT are summarized in Table 3. Unadjusted analyses revealed that the minimum CMT achieved during follow-up was lower in the surgery group (266.40 ± 52.08 μm) compared to the injection group (221.89 ± 38.62 μm), with the difference approaching statistical significance (*p* = 0.051). Both groups exhibited a significant reduction in CMT from their respective baselines (both *p* < 0.01). However, after adjusting for baseline CMT and the number of previous anti-VEGF injections (ANCOVA), a statistically significant difference between the groups was demonstrated. The surgery group maintained a significantly lower adjusted mean minimum CMT (222.67 μm, 95% CI: 189.92–255.42) than the injection group (270.60 μm, 95% CI: 239.70–301.50), with a mean difference of −47.93 μm [95% CI: −95.30 to −0.55; *F*(1, 15) = 4.65, *p* = 0.048].

**TABLE 3:** Comparison of CMT between the two groups.

CMT measure	Surgery group (*n* = 10)	Injection group (*n* = 9)	*t*/*F*-value	*P*-value
**Unadjusted analyses**				
Baseline CMT (μm)	512.50 ± 72.99	461.56 ± 154.60	0.935	0.363
Minimum CMT during follow-up (μm)	266.40 ± 52.08	221.89 ± 38.62	2.095	0.051
Δ CMT (μm)^†^	246.10 ± 93.96	226.11 ± 155.75	0.343	0.736
Within-group *P*-value^‡^	< 0.001***	0.008**		
**Adjusted analysis (ANCOVA)**				
Adjusted minimum CMT (μm) [95% CI]	222.67 [189.92, 255.42]	270.60 [239.70, 301.50]	4.650	0.048*
**Covariates in ANCOVA model**				
Baseline CMT			0.020	0.889
Previous injections			1.202	0.290

Data are presented as mean ± standard deviation unless otherwise indicated. ΔCMT = baseline CMT – minimum CMT during follow-up.

† Unadjusted between-group comparison of ΔCMT by independent *t*-test.

‡ Within-group *P*-values from paired *t*-tests. The model *R*^2^ was 0.324.

*P < 0.05, **P < 0.01, ***P < 0.001.

### 3.4 Edema recurrence condition

After intervention, the recurrence rate of the surgery group was 40%, which was lower than that of the injection group (88.9%, *P* = 0.051). The number of recurrences in the surgery group was less than that in the injection group (*P* = 0.61). Furthermore, among those who experienced recurrence, the surgery group demonstrated a clinically substantial 2.5-fold longer median time to the first recurrence (10.0 months vs. 4.0 months), although this difference was not statistically significant in the log-rank test (*p* = 0.503). At recurrence, CMT increased by 195.50 ± 27.86 μm and 290.00 ± 182.61 μm (*P* = 0.192) in both groups. The peak CMT values achieved were 473.00 ± 45.44 μm and 586.89 ± 169.18 μm (*P* = 0.225) at recurrence, respectively (Table 4). Thickening was lower in the surgery group. Figure 1 shows the full treatment–recurrence–retreatment course of a representative patient from the surgery group.

**TABLE 4:** Comparison of macular edema recurrence between groups.

Recurrence outcome	Surgery group (*n* = 10)	Injection group (*n* = 9)	*P*-value	Effect size (95% CI)
**Recurrence incidence**				
Patients with ≥ 1 recurrence, n (%)	4 (40.0)	8 (88.9)	0.051	OR = 0.08 (0.01, 0.76)
**Time to recurrence**				
Median time to first recurrence (months) [95% CI]	10.0 [0.83–19.17]	4.0 [3.11–4.89]	0.503	HR = 0.68 (0.20, 2.35)
**Burden of recurrence**				
Median number of episodes per patient [IQR]	1 [1, 1.75]	1 [1, 4]	0.61	–
**Severity of recurrence**				
Peak CMT (μm), mean ± SD	473.00 ± 45.44	586.89 ± 169.18	0.225	–
ΔCMT from baseline (μm), mean ± SD	195.50 ± 27.86	290.00 ± 182.61	0.192	–

*P*-values were derived from the following tests: Chi-square test for recurrence incidence; Log-rank test (from Kaplan-Meier analysis) for time to recurrence; Mann-Whitney U test for number of episodes; Independent samples *t*-test for peak CMT and ΔCMT. CI, confidence interval; IQR, interquartile range; CMT, central macular thickness.

### 3.5 Structure-function correlation

To evaluate the concordance between anatomical and functional outcomes, Spearman’s rank correlation analyses were performed on the entire cohort (*n* = 19) (Table 5). No significant correlation was found between baseline BCVA and baseline CMT (ρ = 0.184, *p* = 0.450). Similarly, the correlation between the best postoperative BCVA and the minimum (best) CMT achieved during follow-up was not significant (ρ = 0.224, *p* = 0.356). Most importantly, the correlation between the magnitude of functional improvement (ΔBCVA) and the magnitude of anatomical resolution (ΔCMT, absolute reduction in thickness) showed a negative, non-significant trend (ρ = −0.371, *p* = 0.117), indicating that greater reduction in edema was not strongly associated with greater visual acuity gain in this refractory population.

**TABLE 5:** Structure-function correlations in the refractory RVO-ME (*n* = 19).

Correlation pair	Spearman’s ρ	*P*-value
**Baseline association**		
Preoperative BCVA vs. preoperative CMT	0.184	0.45
**Post-treatment association**		
Best BCVA vs. best CMT	0.224	0.356
**Correlation of improvements**		
ΔBCVA vs. ΔCMT	−0.371	0.117

BCVA, best-corrected visual acuity (logMAR); CMT, central macular thickness (μm); Δ, change from baseline to best post-treatment value.

## 4 Discussion

This is a retrospective study. This study compared the efficacy of surgical and injection treatments for patients with persistent or recurrent edema who had previously received intravitreal injection treatment for RVO-ME. The surgery group was treated with subretinal BSS injection after vitrectomy and ILM peeling. This procedure has not been used in the treatment of RVO-ME in previous studies. We aimed to compare the effectiveness of this procedure with medical therapy in improving visual acuity, resolving edema, and recurrence.

In previous studies (14–17), vitrectomy with or without ILM peeling for the treatment of RVO-ME has improved visual acuity compared with that before surgery, with clear long-term effects. These findings are similar to our unadjusted results. However, after statistical adjustment for baseline imbalances, the difference in final BCVA was no longer significant, suggesting that the observed functional benefit may be influenced by preoperative factors. In the clinic, repeated anti-VEGF therapy is necessary to maintain long-term visual stability and prevent visual loss in patients with RVO-ME (18). In our study, the visual improvement of patients with injection therapy was limited and did not surpass that of surgery after adjustment. In terms of the long-term treatment of RVO-ME, the visual efficacy of anti-VEGF therapy in patients with recurrent edema was reduced (18–20). This may be related to the damage of the retinal structure caused by repeated edema. Second, related to the ceiling effect, patients with poor baseline visual acuity had a better treatment effect than those with better baseline visual acuity.

In our study, vitrectomy combined with internal limiting membrane peeling and subretinal injection of BSS was effective for ME. In our follow-up records, edema resolution was mostly observed at 1 week after surgery. Other studies have mentioned that the possible mechanisms of PPV combined with ILM peeling to improve RVO-ME included the release of traction, removal of angiogenic agents, and improvement of retinal oxygenation (15, 21). In addition, based on the previous application of subretinal BSS injection in DME and severe idiopathic epiretinal membranes (13, 22), we hypothesized that this treatment could dilute the accumulation of inflammatory factors in the retina, increase retinal oxygenation, and promote circulation, thereby improving persistent edema. It is important to note that this mechanistic explanation remains speculative, and the individual contributions of vitrectomy, ILM peeling, and subretinal BSS injection to the overall outcome cannot be disentangled in our study design. In our study, the injection site was chosen based on the 2–3 optic disk diameters from the macula to reduce unnecessary mechanical damage to the macula and ensure that BSS played a therapeutic role in ME.

In terms of recurrence, surgical treatment showed a favorable trend inin recurrence rate and a clinically meaningful extension of the time to recurrence. RVO-ME may be a chronic and long-term condition, and patients with this condition present with recurrent edema that requires long-term repeated anti-VEGF injection therapy for maintenance (23). The mean number of ranibizumab injections up to month 12 is 8.1 (20). Half require at least three injections annually thereafter, and some still need up to six injections in the fourth year. One patient in our injection group received a total of 13 injections for recurrent edema at the later follow-up after three injections of basal loading. (18) If the injection frequency is not sufficient according to the treatment plan, the therapeutic effect is difficult to guarantee. Under pro re nata regimens, an insufficient number of follow-up visits can delay detection of recurrent edema, increasing the risk of undertreatment and subsequent neovascularization (24). The macula undergoes repeated relapses of edema, which damage the photoreceptors. Previous studies have found that significant improvement in retinal thickness is not accompanied by a significant increase in BCVA, and there is no simple linear relationship between retinal thickness and visual acuity (24, 25). This aligns with our findings: while edema in the injection group resolved after retreatment, visual acuity did not improve significantly. Notably, our structure-function correlation analysis confirmed a dissociation in this refractory cohort, showing a negative trend between the magnitude of edema reduction (ΔCMT) and visual gain (ΔBCVA). In contrast, the surgery group demonstrated a more stable treatment effect anatomically. In addition, our study showed that CMT was lower in the surgery group compared with that in the injection group in patients with recurrence. This may be related to the dilution of inflammatory factors by BSS as well as the aforementioned improvement in oxygenation. which helps disrupt the vicious cycle that contributes to chronic ME (13). Surgical intervention thus appears to reduce both the frequency and severity of edema recurrence, providing primarily anatomical benefits that form the foundation for visual stability, improving quality of life, and reducing the burden of repeated treatment.

The safety of subretinal injection of BSS has been confirmed in many studies. In clinical applications the injection can be ensured by controlling the injection dose and pressure using methods similar to our self-made injection device. With the updating of equipment, such as iOCT, the operation of subretinal injections can be visualized. It can assist surgeons with observing the macular area morphology during the injection. The retina bulges rapidly during the injection and the surgeon can judge timely whether there is an iatrogenic macular hole due to pressure on the retina. No macular hole or retinal detachment was observed intraoperatively or postoperatively in our surgical group. In addition, the depth of the needle can be observed using iOCT to avoid damage to the RPE, which affects retinal function. All the above ensure that the technique of subretinal BSS injections can be safely performed during surgery.

In summary, this small, retrospective pilot study provides preliminary evidence for the feasibility of combining PPV, ILM peeling, and subretinal BSS injection for refractory RVO-ME. It provides anatomical advantages in resolving edema and maintaining retinal morphology, with a trend toward visual improvement. This surgical approach may be a valuable adjunctive option in selected cases. A possible limitation is that this study was retrospective and therefore had limited clinical data. Moreover, there is a lack of standardization of anti-VEGF therapy at the time of recurrence. The specific manifestation was that the retreatment of recurrent edema during the follow-up period was not standardized: Multiple anti-VEGF agents (including conbercept and ranibizumab) and steroids (ozurdex). Future prospective, randomized controlled trials with larger cohorts are warranted to validate these preliminary findings and rigorously evaluate its efficacy and safety.

## 5 Conclusion

In conclusion, this retrospective study provides preliminary evidence that the combination of vitrectomy, ILM peeling, and subretinal BSS injection is feasible and significantly associated with anatomical improvement in refractory RVO-ME. These include a reduction in macular foveal thickness as well as a tendency to delay recurrence. While a trend in visual improvement was observed, the observed visual acuity improvement did not reach statistical significance over anti-VEGF therapy after adjustment. These results highlight the potential of this study, and larger prospective randomized trials are needed to confirm these preliminary findings and assess efficacy and safety.

## Data Availability

The raw data supporting the conclusions of this article will be made available by the authors, without undue reservation.
