# Supplementary figures and images for: Subretinal injection of balanced salt solution for macular edema secondary to retinal vein occlusion
Source: Front Med (Lausanne). 2026 Feb 26;13:1701735. doi: 10.3389/fmed.2026.1701735 (PMC12979173; doi:10.3389/fmed.2026.1701735)

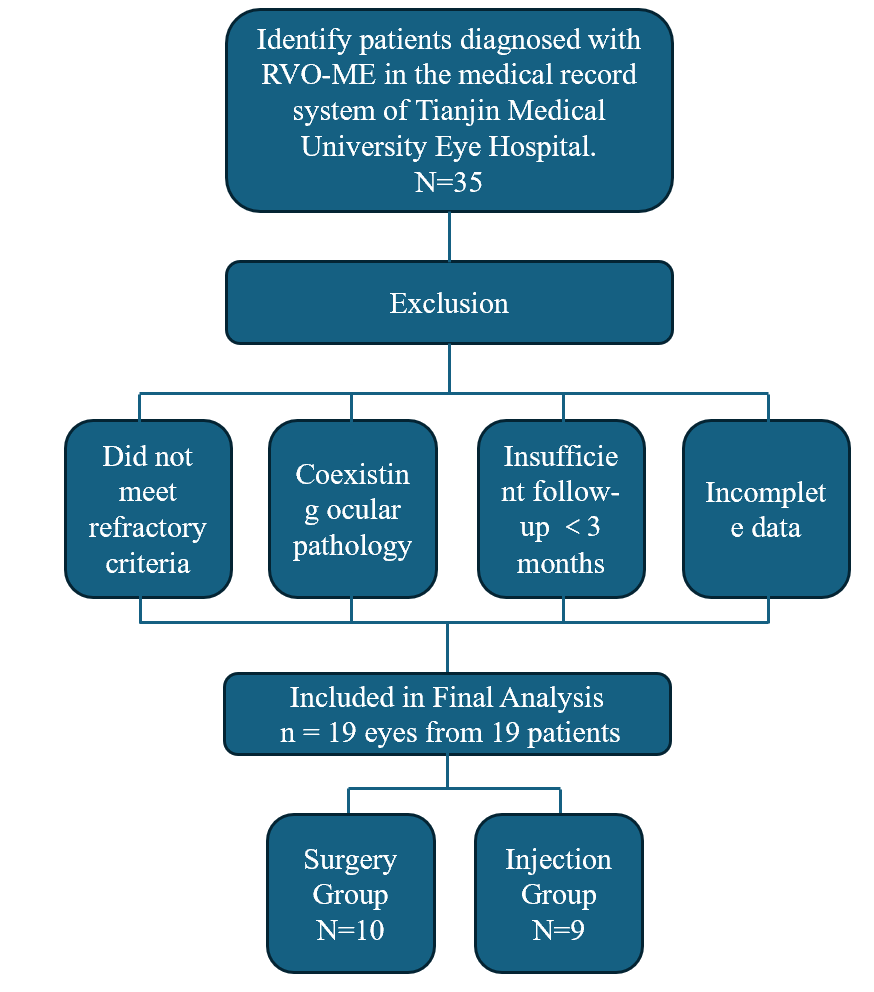

Supplement: Supplementary file 1 [file Table_1.docx]
